# Assessment of different mathematical models for diffusion‐weighted imaging as quantitative biomarkers for differentiating benign from malignant solid hepatic lesions

**DOI:** 10.1002/cam4.1535

**Published:** 2018-05-07

**Authors:** Yao Hu, Hao Tang, Haojie Li, Anqin Li, Jiali Li, Daoyu Hu, Zhen Li, Ihab R. Kamel

**Affiliations:** ^1^ Department of Radiology Tongji Hospital Tongji Medical College Huazhong University of Science and Technology Wuhan Hubei China; ^2^ Russell H. Morgan Department of Radiology and Radiological Science The Johns Hopkins Medical Institutions Baltimore MD USA

**Keywords:** diffusion‐weighted imaging, exponential model, hepatic lesion, magnetic resonance imaging

## Abstract

To quantitatively compare the monoexponential, biexponential, and stretched‐exponential diffusion‐weighted imaging (DWI) models in differentiating benign from malignant solid hepatic lesions. The institutional review board approved this retrospective study and waived the informed consent requirement. A total of 188 patients with 288 hepatic lesions included 202 malignant lesions and 86 benign lesions were assessed (confirmed by pathology or clinical follow‐up for 6 months). All patients underwent hepatic 3.0‐T MRI, including multi‐b DWI that used 12 b values. The ADC,* D*_p_, *D*_t_, perfusion fraction (*f*_p_), α, and DDC values for normal liver, benign liver lesions, and malignant liver lesions were calculated. Independent sample *t* tests were used for comparisons. The diagnostic performance of the parameters was evaluated using ROC analysis. The AUC value for each model was also calculated. The value of *D*_p_ was significantly lower in benign lesions than in normal hepatic parenchyma while others were significantly higher (*P *<* *.001). Whereas Values of *D*_t_ and α in malignant hepatic lesions were significantly higher than in normal hepatic parenchyma (*P *<* *.001), and the *D*_p_ value was significantly lower (*P *<* *.001). Values of ADC,* f*_p_, DDC, and α for malignant hepatic lesions were significantly lower than those for benign hepatic lesions (*P *<* *.001). ROC analysis showed that the diagnostic value of the biexponential model of normal hepatic parenchyma vs benign hepatic lesions and normal hepatic parenchyma vs malignant hepatic lesions was high (0.946 and 0.876, respectively). In the differential diagnosis of benign and malignant hepatic lesions, DDC had the highest AUC value (0.819). The biexponential and stretched‐exponential DWI may provide additional information and improve the differential diagnosis of benign and malignant hepatic lesions compared with the monoexponential DWI.

## 1 BACKGROUND

Hepatic tumors are commonly encountered in clinical practice, and the diagnosis is often not straight forward. A number of studies have shown that the apparent diffusion coefficient (ADC) of monoexponential DWI has the potential to differentiate between benign and malignant focal inflammatory lesion of the liver.^1^, ^2^, ^3^, ^4^, ^5^ However, there is some overlap in ADC values between solid benign and malignant hepatic lesions. The theoretical biexponential model^6^, ^7^, ^8^ provides multiple b values, encompassing both low b values (eg, <200 s/mm^2^) and high b values (>200 s/mm^2^). According to the biexponential model, separate measurement of the perfusion‐related parameters at low b values (perfusion‐related diffusion coefficient *D*_p_ and perfusion fraction *f*_p_) and the pure molecular‐based diffusion coefficient *D*_t_ at high b values can also be obtained with biexponential fitting of the signal intensity versus b curve at multi‐b values using DWI sequences.^6^, ^7^, ^8^

However, the biexponential model is likely an oversimplification of the actual ADC, and it is more realistic to assume a higher number (>2) of intravoxel proton pools with different diffusion coefficients.^9^ To overcome the difficulty of making assumptions about the number of intravoxel proton pools with different diffusion coefficients in biological tissue, Bennett et al^9^, ^10^, ^11^ introduced the stretched‐exponential model. This model introduces new parameters, the distributed diffusion coefficient (DDC), and α value. DDC value represents the mean intravoxel diffusion rate. The α value is a heterogeneity index, and a numerically high α index (ie, α approaching 1) represents low intravoxel diffusion heterogeneity approaching monoexponential decay, while a numerically low α index (ie, α approaching 0) represents a high degree of diffusion heterogeneity exhibited as multi‐exponential decay. Another key point worth emphasizing is that the term “heterogeneity” in this context refers to intravoxel heterogeneity of exponential decay, as opposed to intervoxel heterogeneity of diffusion coefficients, as is often the case in tumors. Thus, the stretched‐exponential and biexponential DWI may be superior to the monoexponential DWI in the differential diagnosis of benign and malignant hepatic lesions.

The aim of this study was to compare the value of monoexponential, biexponential, and stretched‐exponential models with multi‐b‐value DWI in differentiating solid benign from malignant focal hepatic lesions.

## 2 METHOD AND MATERIALS

The institutional review board approved this retrospective study and waived the informed consent requirement.

### 2.1 Study population

**Figure 1:**
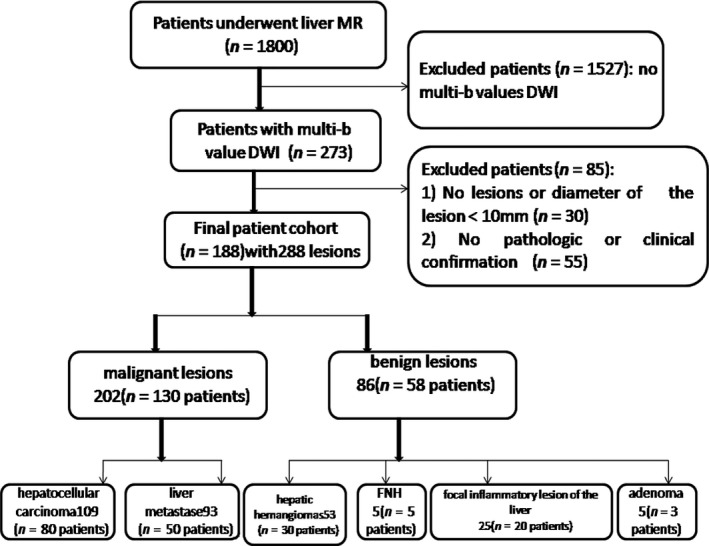
Flowchart of patient selection. FNH, focal nodular hyperplasia

We retrospectively collected the medical records of patients with solid hepatic lesions who had undergone multi‐b values DWI‐MRI scans (273 cases) from the data of all patients receiving a liver MRI scan (1800 cases) between June 2012 and December 2013 at our institution. The inclusion criteria were as follows: (1) patients with suspected or proven one or more solid hepatic lesion; (2) lesion diameter ≥10 mm; and (3) confirmation of hepatic lesions by pathology, by clinical data, or stability on follow‐up imaging for at least 2 years. The study exclusion criteria were as follows: (1) MRI did not show hepatic lesions or was without multi‐b values DWI; (2) lesion largest diameter was <10 mm; or (3) pathology or clinical data were not available or did not confirm the diagnosis. Of the 273 patients, 85 patients were excluded, as seen in Figure 1.

### 2.2 MRI protocol

All patients underwent MRI using a 3.0T MRI scanner (Discovery 750; GE Medical System, Milwaukee, WI, USA) in the supine position. A 32‐channel torso phased‐array coil was used to image the liver. Our routine liver protocol included the following sequences (Table 1): breath‐hold coronal SSFSE T2‐weighted image, transverse in‐phase T1‐weighted image, transverse respiratory‐triggered T2 propeller, and multi‐b‐value DWI.

**Table 1:** MR imaging parameters

Parameter	DWI	T2‐weighted	T1‐weighted
Fat suppression	SPAIR	SPAIR	None
Breath hold	No	No	Yes
acquisition time (s)	303	189	24
Repetition time (ms)/echo time (ms)	5600/112	2982/93	3.4/1.2
Flip angle (degree)	NA	130	11
Section thickness (mm)	6	6	6
Intersection gap(mm)	2	2	2
Bandwidth (Hertz per pixel)	2450	62.5	50
Matrix	160 × 192	320 × 256	320 × 256

NA, not applicable; SPAIR, spectral attenuated inversion recovery.

b Values of 0, 50, 100, 200, 300, 400, 500, 600, 700, 800, 900, and 1000 s/mm^2^ were used for DWI.

Axial respiratory‐triggered single‐shot spin‐echo echo planar sequences were used to acquire DW imaging with the following parameters: TR/TE, 5600/112 ms; matrix size, 160 × 192; b factors of 0, 50, 100, 200, 300, 400, 500, 600, 700, 800, 900, and 1000 s/mm^2^; NEX, 4; slice thickness, 6 mm; and section gap, 2 mm. The diffusion gradient strengths were applied along the X, Y, and Z axes.

### 2.3 Data acquisition and calculation methods

DICOM data from DWI were imported to a workstation with a commercially available software package (ADW4.5; GE Medical Systems) for analysis. Two independent observers, with 5 and 11 years of experience in abdominal radiology, respectively, measured the regions of interest (ROIs). Tumor ADC value was measured three times by each observer by drawing an ROI ≥1 cm within each tumor, and the average of the three measurements was calculated. In addition, each observer replaced ROI ≥1 cm on the background hepatic parenchyma and the average of three measurements was used. Care was used not to include any major vessels within the hepatic parenchyma.

The ADC value was calculated from all 12 b values using a monoexponential model as follows:

S(b)/S(0)=exp(−b·ADC),

where *S*(*b*) represents the signal intensity in the presence of diffusion sensitization and *S*(0) represents the signal intensity in the absence of diffusion sensitization.

Three parameters—perfusion fraction (*f*_p_), tissue diffusivity (*D*_t_), and pseudo diffusivity (*D*_p_)—were calculated using biexponential intravoxel incoherent motion analysis:

$$S\left(b\right)/S\left(0\right)=\left(1-f_{p}\right)\text{exp}\left(-b\;D_{t}\right)+f_{p}\text{exp}\left(-b\;D_{p}\right),$$

where *D*_p_ is the diffusion parameter representing incoherent microcirculation within the voxel (perfusion‐related diffusion, or fast component of diffusion), and *f*_p_ is the fraction of the diffusion linked to microcirculation; *D*_t_ is the true diffusion coefficient that reflects random motion of intra‐ and intercellular water molecules (slow component of diffusion).

Using a stretched‐exponential DWI model, the water molecular diffusion heterogeneity index (α) and the distributed diffusion coefficient (DDC) were obtained by using the following equation:

$$S\left(b\right)/S\left(0\right)=\text{exp}[-{\left(b\cdot \text{DDC}\right)}^{\alpha }],$$

where DDC value represents the mean intravoxel diffusion rate and α is related to the intravoxel water molecular diffusion heterogeneity, which varies between 0 and 1. A numerically high α value represents the low intravoxel diffusion heterogeneity (approaching the monoexponential decay).

**Figure 2:**
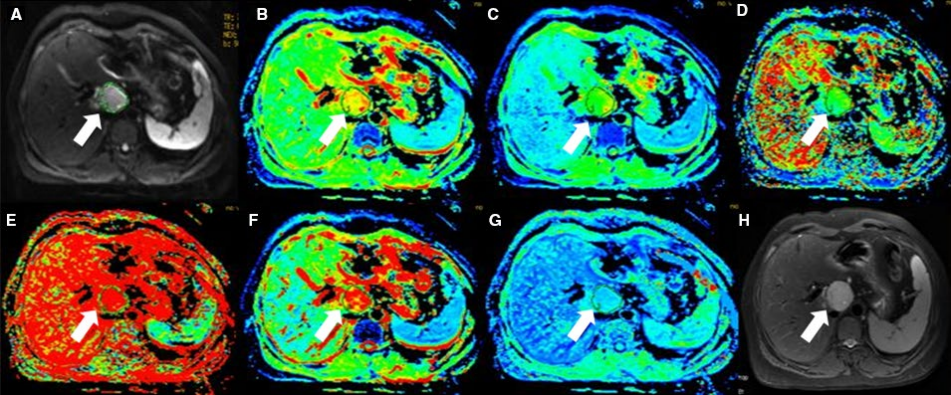
Forty‐five‐year‐old female with hepatic hemangiomas (arrow). (A) is b value of 50 s/mm^2^ of DWI, and (B‐G) are pseudocolor of ADC,* D*_t_, *D*_p_, *f*_p_, DDC and α. The values of lesion were 1.9 × 10^−3^, 1.45 × 10^−3^, 1.41 × 10^−2 ^mm^2^/s, 0.318, 2.35 × 10^−3 ^mm^2^/s, and 0.635, respectively. (H) is T2 image

**Figure 3:**
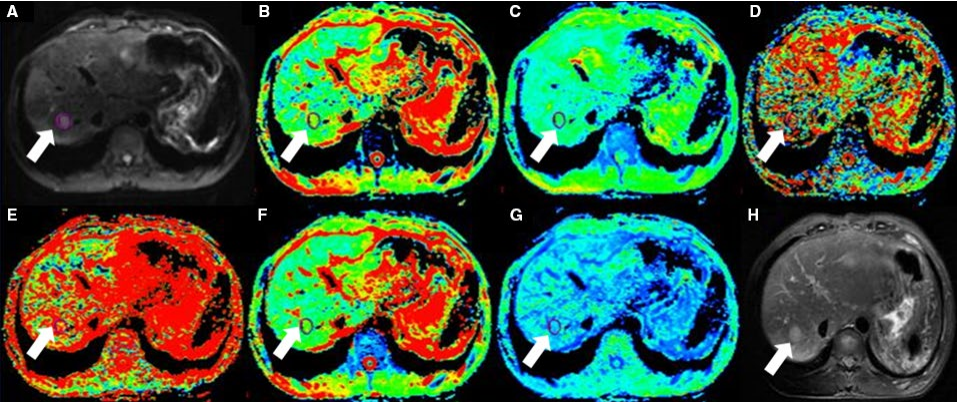
Fifty‐six‐year‐old male with HCC(arrow). (A) is b value of 50 s/mm^2^ of DWI, and (B‐G) are pseudocolor of ADC,* D*_t_, *D*_p_, *f*_p_, DDC, and α. The values of lesion were 1.45 × 10^−3^, 1.02 × 10^−3^, 3.12 × 10^−2 ^mm^2^/s, 0.25, 1.52 × 10^−3 ^mm^2^/s, and 0.567, respectively. (H) is T2 image

**Figure 4:**
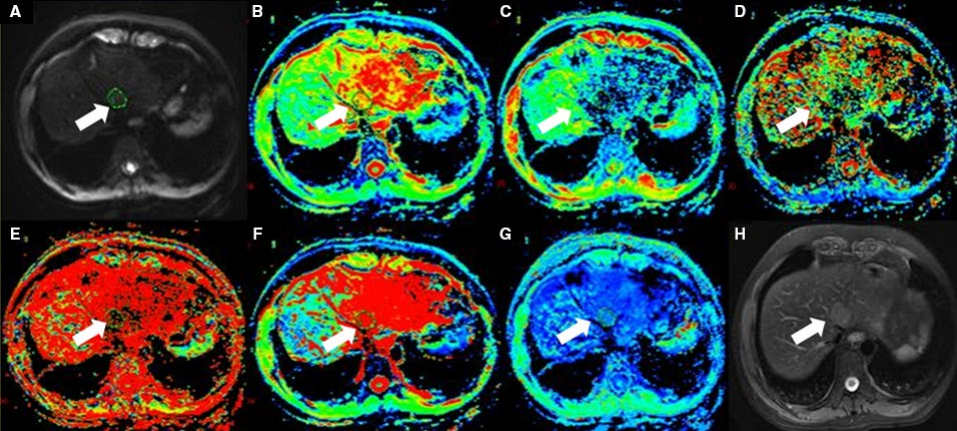
Thirty‐two‐year‐old male with hepatic inflammatory nodules(arrow). (A) is b value of 50 s/mm^2^ of DWI, and (B‐G) are pseudocolor of ADC,* D*_t_, *D*_p_, *f*_p_, DDC and α. The values of lesion were 2.04 × 10^−3^, 0.344 × 10^−3^, 1.3 × 10^−2 ^mm^2^/s, 0.67, 5.07 × 10^−3 ^mm^2^/s, and 0.414, respectively. (H) is T2 image

**Figure 5:**
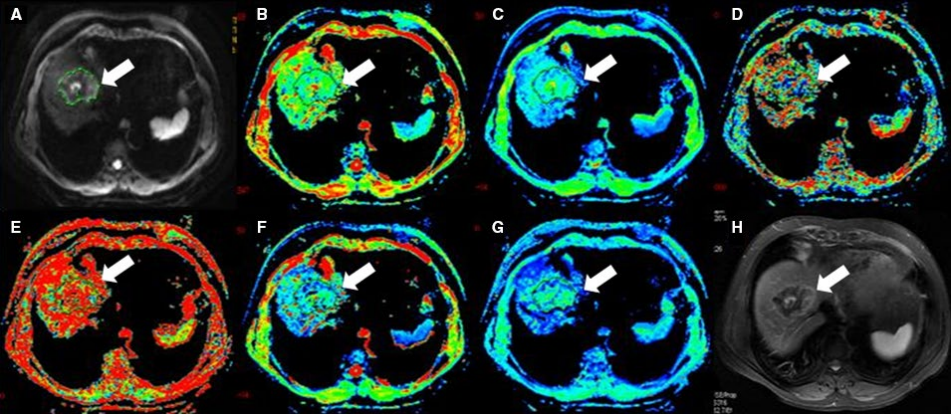
Seventy‐two‐year‐old female with liver metastases(arrow). (A) is b value of 50 s/mm^2^ of DWI, and (B‐G) are pseudocolor of ADC,* D*_t_, *D*_p_, *f*_p_, DDC and α. The values of lesion were 0.923 × 10^−3^, 0.807 × 10^−3^, 1.44 × 10^−2 ^mm^2^/s, 0.345, 0.608 × 10^−3 ^mm^2^/s, and 0.702, respectively. (H) is T2 image

ADC, *D*_t,_ *D*_p_, *f*_p_ fraction, DDC and α value were automatically generated by the software (Figures 2, 3, 4, 5).

### 2.4 Statistical analyses

Statistical processing and analysis of the data were performed using statistical software (IBM SPSS for Windows, version 18.0; SPSS, Chicago, IL, USA). All solid hepatic lesions were classified as either benign or malignant. The data points were compared using an independent sample *t* test for statistical analysis. *P* values <.05 indicated statistical significance. In differentiating among the normal hepatic parenchyma, benign lesions, and malignant lesions, each of the parameter values in the three exponential models was considered separately for the ROC curve analysis and the comparison of the area under the curve (AUC). Combined AUC was also calculated for of the biexponential model and the stretched‐exponential model.

## 3 RESULTS

### 3.1 Patient demographics

The final cohort included 188 patients (125 men and 63 women, age range of 19‐79 years) with 288 lesions, including 202 malignant lesions (109 hepatocellular carcinoma and 93 hepatic metastases) and 86 benign lesions (53 hepatic hemangiomas, 25 focal inflammatory lesion of the liver (FILLs), five focal nodular hyperplasia, and three adenoma) (Table 2).

**Table 2:** Patient demographics

Patient group	No. of patients	Mean age (years)	Benign hepatic lesions	Malignant hepatic lesions
Men	125	56	53	109
Women	63	55	25	93

### 3.2 Monoexponential, biexponential, and stretched‐exponential DWI analysis in normal hepatic parenchyma and in benign and malignant hepatic lesions

**Figure 6:**
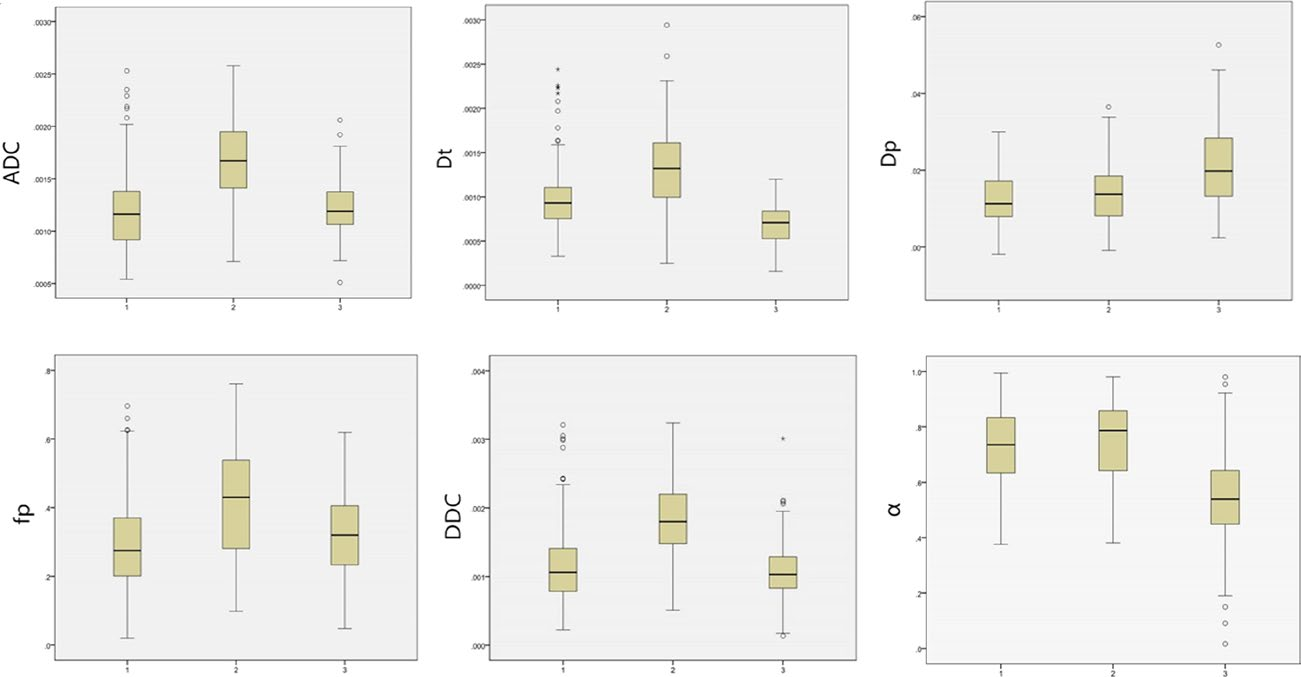
Box plots of parameters for normal hepatic parenchyma, benign, and malignant hepatic lesions. 1 = malignant hepatic lesions, 2 = benign hepatic lesions, 3 = normal hepatic parenchyma

Values and ranges of ADC, *D*_t_, *D*_p_, *f*_p_, DDC, and α in normal hepatic parenchyma, benign hepatic lesions, and malignant hepatic lesions are described in Table 3 and Figure 6. The ADC value of normal hepatic parenchyma was significantly lower than that of benign hepatic lesions (*P *<* *.001) but was not different from that of malignant hepatic lesions (*P *=* *.522). The ADC value of benign hepatic lesions was significantly lower than that of malignant hepatic lesion (*P *<* *.001).

**Table 3:** Mean values of measured parameters for normal liver parenchyma, benign, and malignant hepatic lesions

Parameter	Normal liver parenchyma	Benign hepatic lesions	Malignant hepatic lesions	*P*	*P*1	*P*2
ADC (×10^−3^ mm^2^/s)	1.18 ± 0.26	1.67 ± 0.39	1.21 ± 0.4	<.001	.522	<.001
*D*_t_ (×10^−3^ mm^2^/s)	0.68 ± 0.22	1.32 ± 0.49	1.13 ± 0.89	<.001	<.001	.083
*D*_p_ (×10^−2^ mm^2^/s)	2.19 ± 1.24	1.44 ± 0.8	1.32 ± 0.73	<.001	<.001	.212
*f*_p_	0.32 ± 0.12	0.41 ± 0.18	0.29 ± 0.14	<.001	.053	<.001
DDC (×10^−3^ mm^2^/s)	1.41 ± 1.53	2.2 ± 1.37	1.29 ± 0.99	<.001	.407	<.001
α	0.55 ± 0.17	0.81 ± 0.18	0.8 ± 0.19	<.001	<.001	.600

Data are mean ± SD. *P* is normal hepatic parenchyma vs benign hepatic lesions**,** *P*1 is normal hepatic parenchyma vs malignant hepatic lesions**,** *P*2 is benign hepatic lesions vs malignant hepatic lesions. The *f*_p_ value is the fraction of *D*_p_, which represents a perfusion‐related percentage. The α value represents the heterogeneity within the voxel size and ranges from 0 to 1.

Using the exponential model, the *D*_t_ value of normal hepatic parenchyma was significantly lower than that of benign and malignant hepatic lesions (*P *<* *.001 for both). However, the *D*_t_ value of benign and malignant hepatic lesions was not significant (*P *=* *.083). The *D*_p_ value of normal hepatic parenchyma was significantly higher than that of benign and malignant hepatic lesions (*P *<* *.001 for both). However, the *D*_p_ value of benign and malignant hepatic lesions was not significant (*P *=* *.212). In addition, although the *f*_p_ value of normal hepatic parenchyma was not significantly different from that of malignant hepatic lesions (*P *=* *.053), it was significantly lower than that of benign hepatic lesions (*P *<* *.001). The *f*_p_ value of benign and malignant hepatic lesions was significantly different (*P *<* *.001).

In the stretched‐exponential model, the DDC value of normal hepatic parenchyma was significantly lower than that of benign hepatic lesions (*P *<* *.001), and the DDC value of malignant hepatic lesions was significantly lower than that of benign hepatic lesions (*P *<* *.001). However, the DDC value of normal hepatic parenchyma was not significantly different from that of malignant hepatic lesions (*P *=* *.407). The α value of normal hepatic parenchyma was significantly lower than that of benign and malignant hepatic lesions (*P *<* *.001 for both). However, α value of benign and malignant hepatic lesions was not different (*P *=* *.600).

### 3.3 ROC analysis

Using ROC analysis, the differentiation of normal hepatic parenchyma from benign hepatic lesions on the monoexponential model had AUC of 0.833. The combined AUC value of the three parameters of the biexponential DWI (*D*_t_, *D*_p_, and *f*_p_) and the two parameters of the stretched‐exponential DWI (DDC and α) was 0.946 and 0.828, respectively (Table 4). In the differentiation between normal hepatic parenchyma and malignant hepatic lesions, the AUC of α had the highest value (0.825). However, the combined AUC of the biexponential DWI was higher (0.876 (Table 4). For the differentiation between benign and malignant hepatic lesions, DDC showed the highest AUC (0.819) compared with AUC of other parameters. The AUC value of the stretched‐exponential DWI was also high (0.819) (Table 4).

**Table 4:**
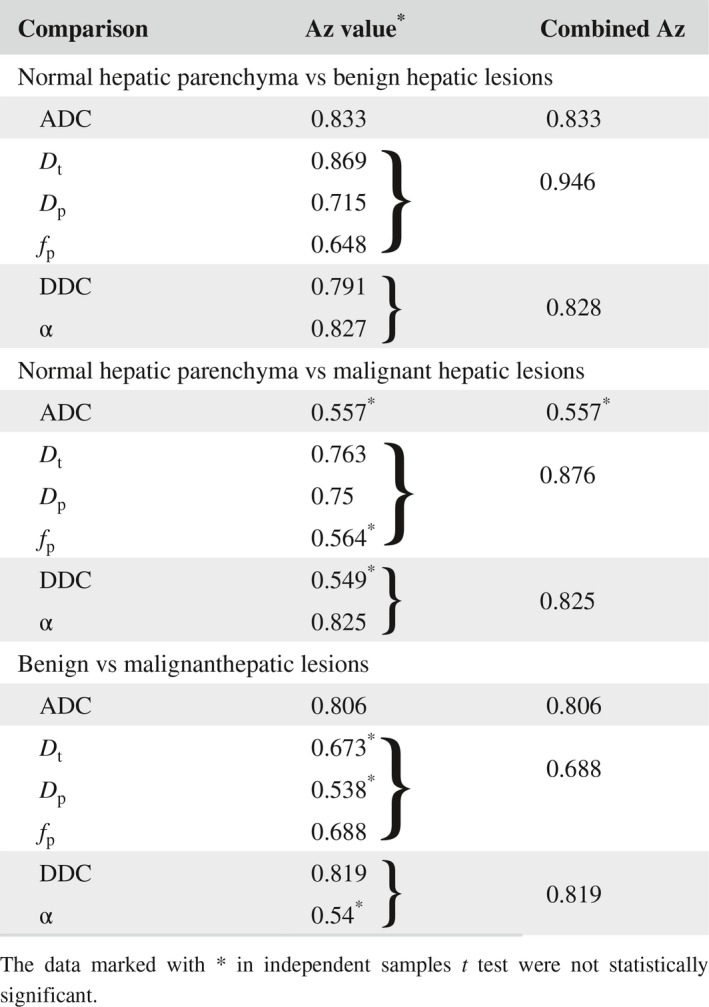
Results of ROC analysis for multi‐b DWI parameters

## 4 DISCUSSION

The results of this study showed that utilizing the monoexponential DWI, the ADC value of benign hepatic lesions was significantly higher than that of normal hepatic parenchyma (Table 3, *P *<* *.001), which is consistent with the results of previous studies.^12^, ^13^, ^14^, ^15^, ^16^, ^17^ However, the results of this study also showed that the ADC values of malignant hepatic lesions and normal hepatic parenchyma were not statistically significant (Table 3, *P *=* *.522), which is inconsistent with the results of previous studies. This may be related to the different malignant lesions included in this study, including primary and metastatic disease. In this study, hepatic malignant lesions included hepatic metastases and HCC. The mean ADC of HCC (1.12 ± 0.32 × 10^−3^ mm^2^/s) is lower than normal parenchyma (1.18 ± 0.26 × 10^−3^ mm^2^/s) (*P *< .05), however, hepatic metastases including many types of sources. There may be mucous, cystic, and necrotic in hepatic metastases. The mean ADC of hepatic metastases (1.31 ± 0.46 × 10^−3^ mm^2^/s) is higher than normal parenchyma (1.18 ± 0.26 × 10^−3^ mm^2^/s) (*P *< .05). As a result, the mean ADC of malignant lesions (1.21 ± 0.4 × 10^−3^ mm^2^/s) is not different from normal parenchyma in our study(1.18 ± 0.26 × 10^−3^ mm^2^/s) (*P *> .05).

Using biexponential DWI our study showed that the *D*_t_ values of benign and malignant hepatic lesions were higher than those of normal hepatic parenchyma (Table 3, *P *< .001). This may indicate that both benign and malignant lesions had significantly accelerated diffusion. The *D*_p_ value of benign and malignant hepatic lesions was lower than that of normal hepatic parenchyma, indicating that the extracellular space associated with benign hepatic lesions and malignant tissue was significantly limited, thereby limiting the diffusion of cellular water molecules and resulting in a significantly reduced *D*_p_ value. Therefore, the *D*_t_ and *D*_p_ value could differentiate normal hepatic parenchyma from benign and from malignant hepatic lesions, but could not differentiate benign from malignant hepatic lesions, which is inconsistent with the results of a previous study.^18^ The reason may be that hepatic metastases including many types of sources in this study. There are mucous, cystic, and necrotic in hepatic metastases. These factors will have an impact on *D*_t_ and *D*_p_ value. The *f*_p_ value of benign hepatic lesions was higher than that for normal hepatic parenchyma and malignant hepatic lesions, but there was no significant difference between the *f*_p_ value of normal hepatic parenchyma and malignant lesions. The *f*_p_ is the fraction of the diffusion linked to microcirculation. This result indicated that the change of microcirculation of malignant hepatic lesions is not significant. However, the *f*_p_ value was able to identify normal hepatic parenchyma from benign hepatic lesions and benign from malignant hepatic lesions. The possible reason is that the microcirculation of benign hepatic lesions changes significantly.

In this study, using the stretched‐exponential DWI, the DDC value of benign hepatic lesions was higher than that of normal hepatic parenchyma and malignant hepatic lesions (Table 3, *P *< .001), but was not significantly different between normal liver parenchyma and malignant hepatic lesions (Table 3, *P *> .05). The average intravoxel diffusion of benign hepatic lesions was higher than that of normal liver parenchyma and average intravoxel diffusion of malignant hepatic lesions was not different from that of normal hepatic parenchyma. Thus the DDC value could distinguish between normal hepatic parenchyma and benign hepatic lesions and between benign and malignant hepatic lesions, but could not distinguish between normal hepatic parenchyma and malignant hepatic lesions. DDC value represents the mean intravoxel diffusion rate. It has the characteristics of the standard diffusion coefficient. It can be considered as a compound parameter of the continuous distribution part of each ADC weighted by the volume fraction of water molecules. Therefore, the result of DDC is the same as ADC. The parameter of α is related to the intravoxel water molecular diffusion heterogeneity. The “heterogeneity” here refers to the heterogeneity of the exponential decay in the voxels, rather than the heterogeneity of the diffusion coefficient. There are more cell components in both benign and malignant hepatic lesions than normal hepatic parenchyma. And then the signal attenuation of voxels in hepatic tumors is relative consistent. The α value of both benign and malignant hepatic lesions was higher than in normal hepatic parenchyma, indicating lower voxel diffusion heterogeneity of hepatic tumors. Thus, the α value could distinguish normal hepatic parenchyma from benign and malignant hepatic lesions, but not between benign and malignant hepatic lesions.

In the current study, the combined AUC value of the biexponential DWI was significantly higher than the AUC value of the monoexponential and stretched‐exponential DWI in distinguishing normal hepatic parenchyma from benign hepatic lesions. The highest AUC value for distinguishing normal hepatic parenchyma from malignant hepatic lesions was for α. Thus, compared to normal hepatic parenchyma, the diffuse heterogeneity within the voxels of malignant lesions was significantly increased. However, the stretched‐exponential model, which includes both the DDC and α, did not result in improved accuracy in distinguishing between normal hepatic parenchyma and malignant hepatic lesions. However, the AUC of the biexponential DWI which included *D*_t_ and *D*_p_ and *f*_p_ combined curve (0.876) was significantly higher than that of α (0.825). This result may be due to the incremental value of the three parameters compared to a single parameter. As a result, the biexponential DWI has the highest accuracy in distinguishing normal hepatic parenchyma from malignant hepatic lesions.

In the current study, DDC had the highest value (0.819) in distinguishing between benign and malignant hepatic lesions. This suggests that benign hepatic lesions have a larger average diffusion rate than malignant lesions. Thus, the stretched‐exponential DWI may have the highest accuracy in the distinction between benign and malignant hepatic lesions.

Certain limitations of this study should be noted. Firstly, although the multi‐b values for DWI were respiratory triggered, the image quality was degraded by cardiac pulsation, resulting in limited measurements, especially affecting small lesions in the left hepatic lobe. This was also described in prior studies.^19^, ^20^ Secondly, the number of study patients was relatively small. Further prospective analyses of a larger number of patients will be needed to validate our results. Lastly, we did not stratify hepatic metastases into those with a rich blood supply and poor blood supply, and the ADC values of these two types may be different. Further in‐depth study is needed to address this issue.

## 5 CONCLUSIONS

In conclusion, the biexponential DWI model had the highest AUC value in distinguishing between normal hepatic parenchyma and benign hepatic lesions, and between normal hepatic parenchyma and malignant hepatic lesions. The stretched‐exponential model had the highest AUC value in distinguishing benign from malignant hepatic lesions.

## COMPETING INTERESTS

The authors declare that they have no competing interests.

## DECLARATIONS

Ethics approval and consent to participate: The institutional review board of the clinical trial ethics committee of Huazhong University of science and technology approved this retrospective study and waived informed consent. The approval number is S097.

## CONSENT FOR PUBLICATION

Not applicable.

## AVAILABILITY OF DATA AND MATERIAL

The datasets used and/or analyzed during the current study are available from the corresponding author on reasonable request.
